# Supplementary material for: Integration of Selection Signatures and Protein Interactions Reveals NR6A1, PAPPA2, and PIK3C2B as the Promising Candidate Genes Underlying the Characteristics of Licha Black Pig
Source: Biology (Basel). 2023 Mar 25;12(4):500. doi: 10.3390/biology12040500 (PMC10135650; doi:10.3390/biology12040500)

biology

Supplementary Materials

Integration of Selection Signatures and Protein Interactions Reveals *NR6A1, PAPPA2,* and *PIK3C2B* as the Promising Candidate Genes Underlying the Characteristics of Licha Black Pig

Figure S1. Distribution of the sequenced SNPs on all autosome. The x-axis represents the corresponding chromosomal position (Mb), and the y-axis represents chromosomes. Different colors of each 1Mb genome block denote the number of SNPs.

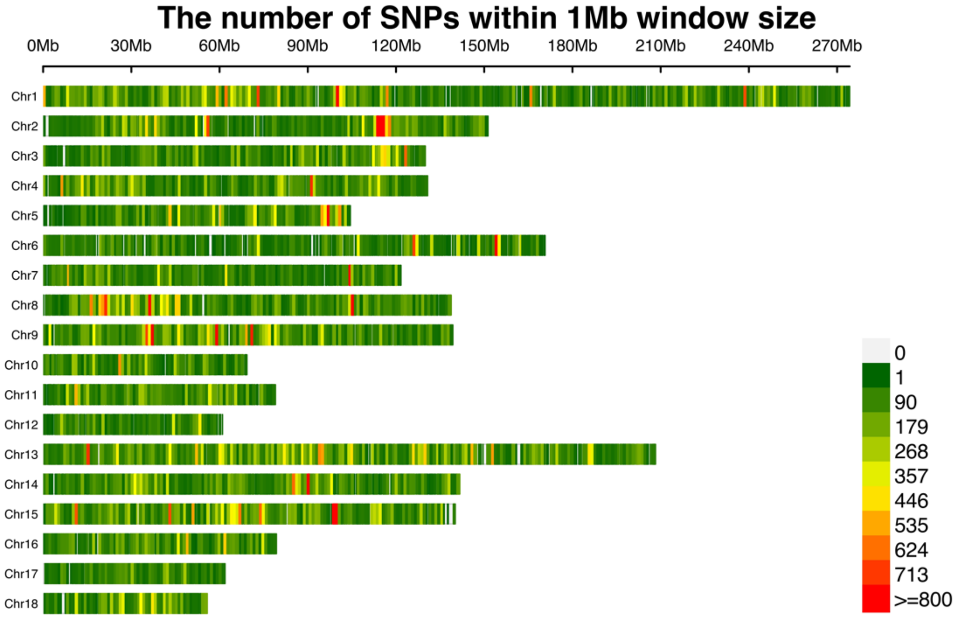

Figure S2. Population structures were inferred using ADMIXTURE with the assuming number of ancestral cluster K from 1 to 20.

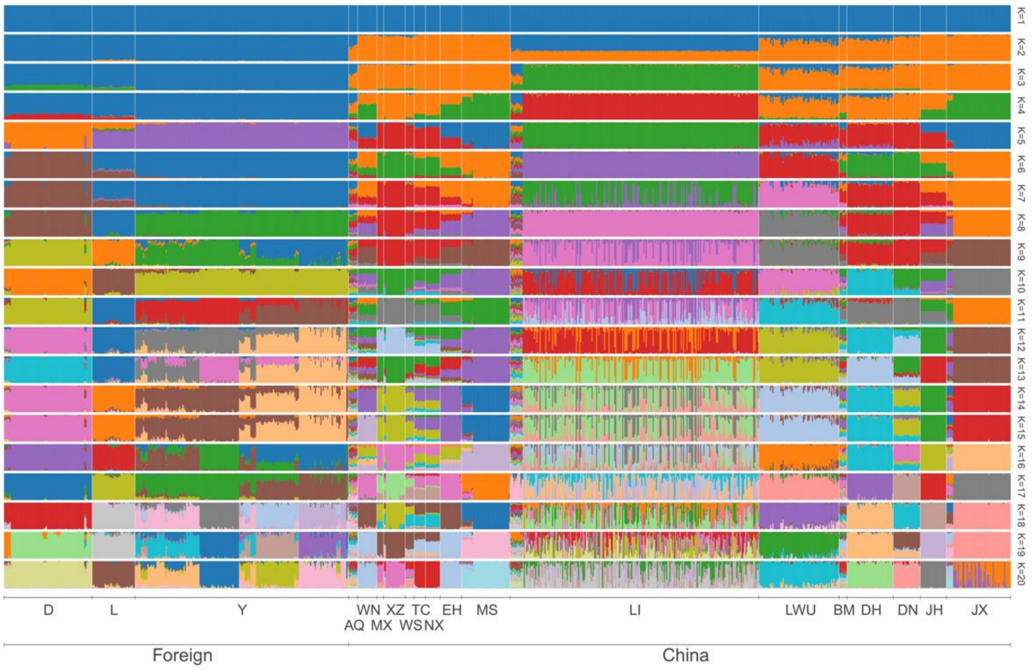

Figure S3. The number and coverage of ROH fragments on each chromosome. The x-axis represents chromosomes, the left y-axis represents the total number of runs of homozygosity (ROH) segments, and the right y-axis represents the coverage of ROH segments.

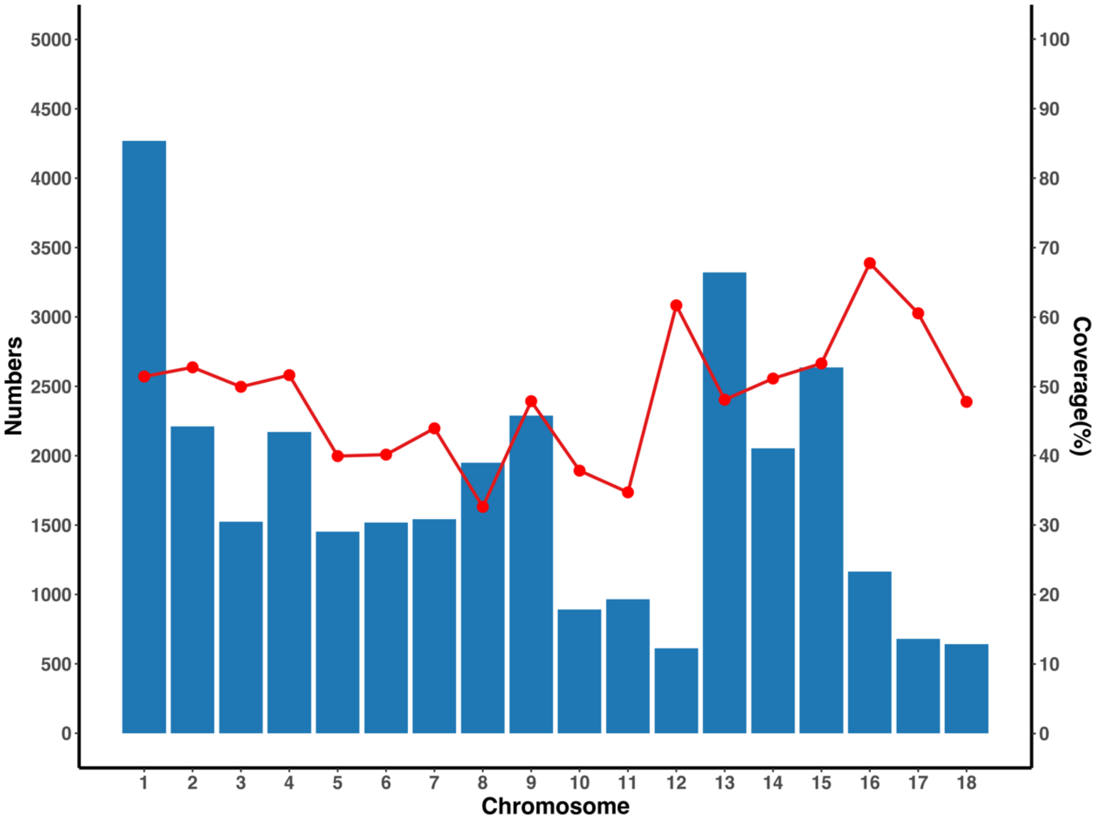

Figure S4. The number and length of ROH of all individuals in each breed. The x-axis represents the total number of runs of homozygosity (ROH) fragments for all individuals from each breed, and the y-axis represents the total length (Mb) of genome in such ROH for all individuals from each breed. Each dot represents an individual.

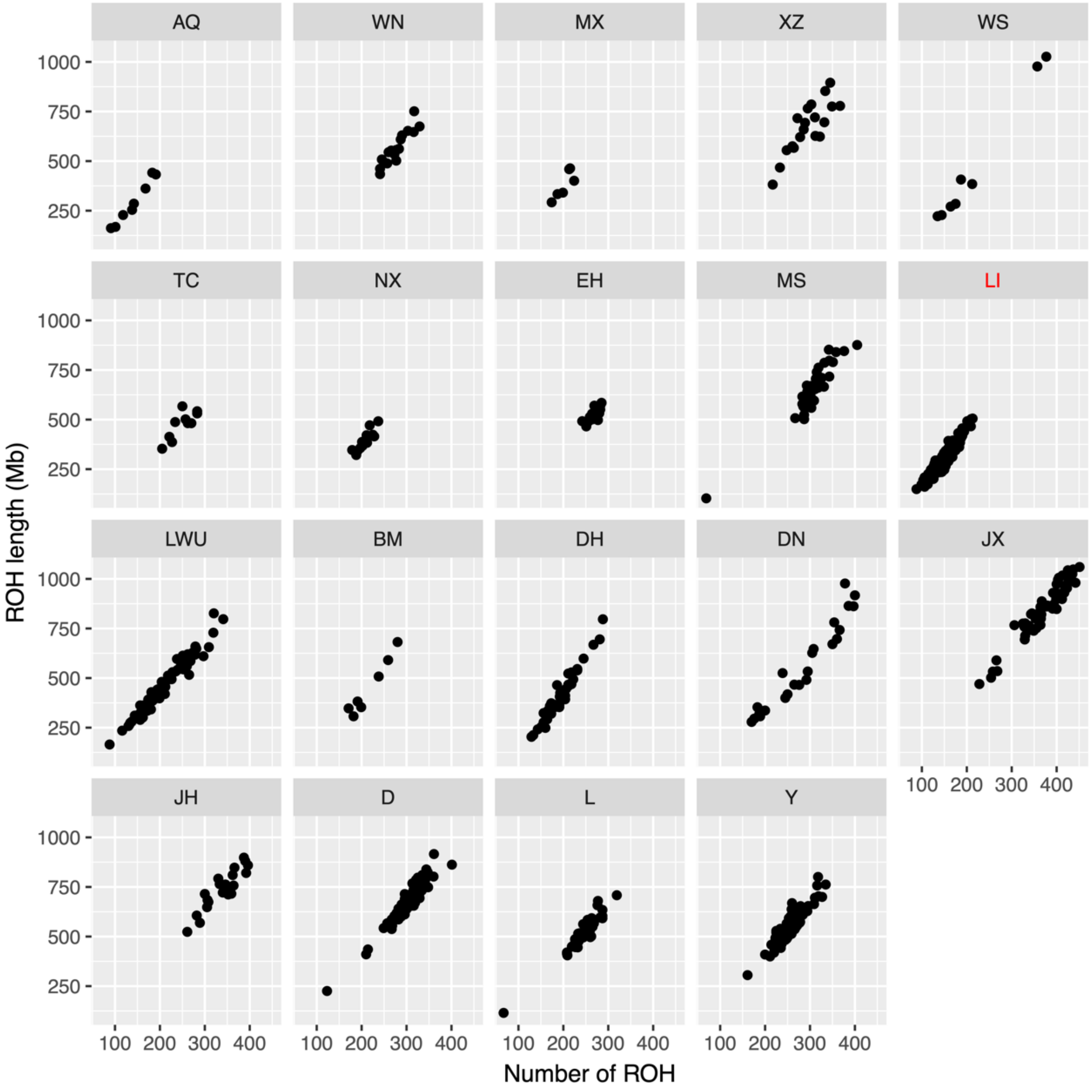

Figure S5. Venn diagram of the number distribution of candidate genes within LI pigs by three methods (ROH, haplotype and F_{ST}). FST-1, the F_{ST} between LI pigs and other Chinese pigs. FST-2, the F_{ST} between LI pigs and commercial pigs.

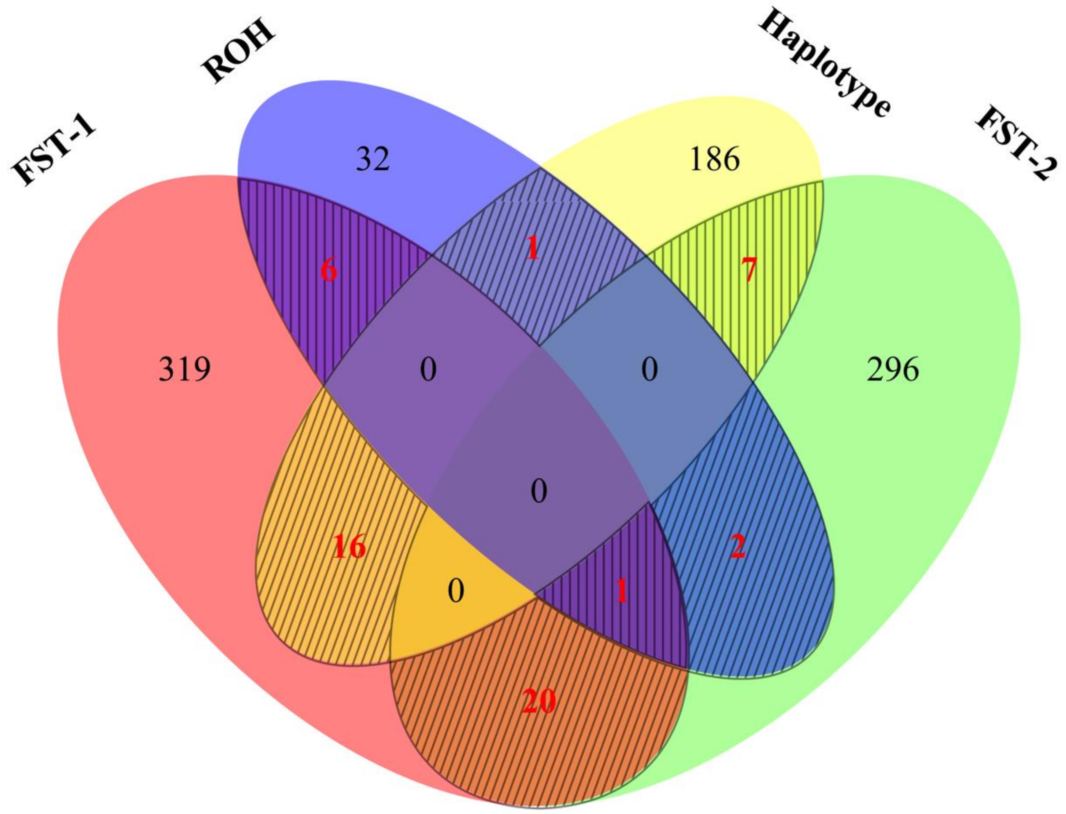

Supplement: Supplementary file 1 [file biology-12-00500-s001.zip › biology-2211574- Supplementary Figures.pdf]
